# Impact of loss of BH3-only proteins on the development and treatment of *MLL*-fusion gene-driven AML in mice

**DOI:** 10.1038/cddis.2016.258

**Published:** 2016-09-01

**Authors:** Rebecca A Bilardi, Natasha S Anstee, Stefan P Glaser, Mikara Robati, Cassandra J Vandenberg, Suzanne Cory

**Affiliations:** 1 Walter and Eliza Hall Institute of Medical Research, Melbourne, Victoria 3052, Australia; 2 Department of Medical Biology, University of Melbourne, Victoria 3010, Australia

## Abstract

Inhibition of the apoptosis pathway controlled by opposing members of the Bcl-2 protein family plays a central role in cancer development and resistance to therapy. To investigate how pro-apoptotic Bcl-2 homology domain 3 (BH3)-only proteins impact on acute myeloid leukemia (AML), we generated mixed lineage leukemia (*MLL)-AF9* and *MLL-ENL* AMLs from BH3-only gene knockout mice. Disease development was not accelerated by loss of Bim, Puma, Noxa, Bmf, or combinations thereof; hence these BH3-only proteins are apparently ineffectual as tumor suppressors in this model. We tested the sensitivity of *MLL-AF9* AMLs of each genotype *in vitro* to standard chemotherapeutic drugs and to the proteasome inhibitor bortezomib, with or without the BH3 mimetic ABT-737. Loss of Puma and/or Noxa increased resistance to cytarabine, daunorubicin and etoposide, while loss of Bim protected against cytarabine and loss of Bmf had no impact. ABT-737 increased sensitivity to the genotoxic drugs but was not dependent on any BH3-only protein tested. The AML lines were very sensitive to bortezomib and loss of Noxa conveyed significant resistance. *In vivo,* several *MLL-AF9* AMLs responded well to daunorubicin and this response was highly dependent on Puma and Noxa but not Bim. Combination therapy with ABT-737 provided little added benefit at the daunorubicin dose trialed. Bortezomib also extended survival of AML-bearing mice, albeit less than daunorubicin. In summary, our genetic studies reveal the importance of Puma and Noxa for the action of genotoxics currently used to treat MLL-driven AML and suggest that, while addition of ABT-737-like BH3 mimetics might enhance their efficacy, new Noxa-like BH3 mimetics targeting Mcl-1 might have greater potential.

Acute myeloid leukemia (AML) is a devastating disease primarily affecting children and older people. Although genetically diverse,^1, 2^ most AMLs are oligoclonal at presentation, with perhaps only two to four driver mutations.^1, 3, 4, 5^ Chromosomal translocations are common (~50% of cases) and those involving the mixed lineage leukemia (*MLL*) gene, the mammalian homolog of *Drosophila* trithorax gene located on chromosome 11 band q23, occur in ~10% of acute leukemias, including AML, acute lymphoblastic leukemia and leukemias of mixed or indeterminate lineage.^6^ *MLL* translocations are associated with poor prognosis.^6, 7^ *MLL* encodes a large multi-domain protein that activates transcription through its C-terminal histone H3 lysine 4 (H3K4) methyl transferase domain. *MLL* translocations create a fusion gene containing the 5′ portion of *MLL* and the 3′ portion of the partner gene.^8^ The DNA-binding MLL portion of the resulting fusion protein binds MLL target genes, including Hox genes, and the partner moiety enforces constitutive expression through interaction with a higher order transcriptional elongation complex.^6, 7, 9^ Nearly 80 different MLL fusion partners have been identified in AML,^7^ two of the most common being *AF9* and *ENL*. Transgenic mice expressing *Mll*-*AF9* and *Mll-ENL* under the control of the endogenous *mll* promoter are highly prone to AML, although the long latency indicates a requirement for additional genetic event(s) before the emergence of fully malignant cells.^10, 11, 12^

Major improvements in AML therapy have remained elusive. Current ‘standard of care' involves an initial phase of intense chemotherapy (remission induction therapy) followed by additional chemotherapy cycles and/or allogeneic stem cell transplantation. Most commonly, induction therapy involves administration of cytarabine with an anthracycline, usually daunorubicin or idarubicin, with etoposide sometimes also included. Because all these drugs act on DNA synthesis, they preferentially affect rapidly dividing cells. Cytarabine (cytosine arabinoside) is phosphorylated intracellularly and incorporated into DNA during S-phase, resulting in chain termination of the elongating nascent DNA chain.^13^ Anthracyclines and etoposide inhibit topoisomerase II, thereby increasing the frequency of double strand DNA breaks.^14^ Multiple additional activities have been ascribed to anthracyclines,^15^ including inhibition of DNA and RNA synthesis as a result of intercalation between base pairs and generation of damaging reactive oxygen species (ROS).^16^

By provoking DNA damage, ROS and other intracellular stresses, cytotoxic drugs kill cells (at least in part) by inducing the intrinsic (also known as the mitochondrial or stress-induced) apoptosis pathway, which is regulated by pro- and anti-apoptotic members of the Bcl-2 family (for reviews see refs 17, 18, 19). Bcl-2 and its closest relatives (Bcl-x_L_, Mcl-1, A1/BFL1, Bcl-w and, in humans, possibly also Bcl-B) promote cell survival by inhibiting apoptosis, whereas structurally similar relatives Bax and Bak (and possibly also Bok) instead promote apoptosis, as do the so-called ‘BH3-only proteins' (Bim, Puma, Noxa, Bad, Bid, Bmf, Bik and Hrk), which have only one of the four Bcl-2 homology (BH) domains. In healthy cells, the pro-survival proteins hold Bax and Bak in check. Stress signals – such as DNA damage or oncogene expression – up-regulate expression of Bcl-2 homology domain 3 (BH3)-only proteins, which bind tightly to the hydrophobic surface groove of pro-survival Bcl-2-like proteins, thereby neutralizing their capacity to inhibit activated Bax and Bak. The most potent BH3-only proteins, Bim, Puma and tBid, can bind all pro-survival proteins whereas others show more selective binding:^20, 21^ Noxa, for example, is specific for Mcl-1 and A1/BFL1, whereas Bad is specific for Bcl-2, Bcl-x_L_ and Bcl-w. Certain BH3-only proteins can also bind transiently to the surface groove of Bax and Bak, inducing them to undergo conformational change and form homodimers on the outer mitochondrial membrane.^22, 23^ The homodimers then aggregate to form homo-oligomeric pores,^24^ through which cytochrome *c* egresses into the cytoplasm to initiate the cascade of caspase activation responsible for dismantling the cell.

A new class of small molecule that mimics BH3-only proteins is generating much clinical interest. BH3 mimetics bind to pro-survival Bcl-2-like proteins in a manner similar to BH3-only proteins, releasing previously sequestered BH3-only proteins to activate Bax and Bak.^25, 26^ Cancer cells have greater susceptibility to BH3 mimetic drugs than normal cells, in part because they often have higher levels of pro-survival proteins and hence higher ‘stores' of BH3-only proteins.^26, 27^ ABT-737, the first bonafide BH3 mimetic to be developed, binds Bcl-2, Bcl-x_L_ and Bcl-w but not Mcl-1 or A1,^28, 29^ as does its orally bio-available derivative ABT-263 (Navitoclax),^30^ whereas the more recent ABT-199 (Venetoclax) is specific for Bcl-2 (ref. 31) and A-1155463 is specific for Bcl-x_L_.^32^ These BH3 mimetics have shown promising efficacy in a variety of preclinical models (eg refs 31, 33) and ABT-263 and ABT-199 are now in advanced clinical trials for chronic lymphocytic leukemia and certain other malignancies.^34, 35, 36, 37^ High levels of Mcl-1 (or A1/BFL1) are likely to cause resistance^26, 29, 38^ and BH3 mimetics specific for Mcl-1 are under development.

Using retroviruses expressing MLL-fusion genes,^39, 40, 41^ we are generating murine AMLs with a variety of apoptotic lesions. Here we report genetic studies designed to clarify whether BH3-only proteins act as tumor suppressors during AML development and which of them are critical for the response of MLL fusion gene-driven AMLs to standard chemotherapeutics (cytarabine, daunorubicin and etoposide) or to the proteasome inhibitor bortezomib, which is being trialed clinically for AML.^42, 43^ We also tested whether the MLL AMLs are sensitive to ABT-737, and whether combination therapy with ABT-737 improves sensitivity.

## Results

### Generation of murine AMLs lacking specific BH3-only proteins

**Figure 1:**
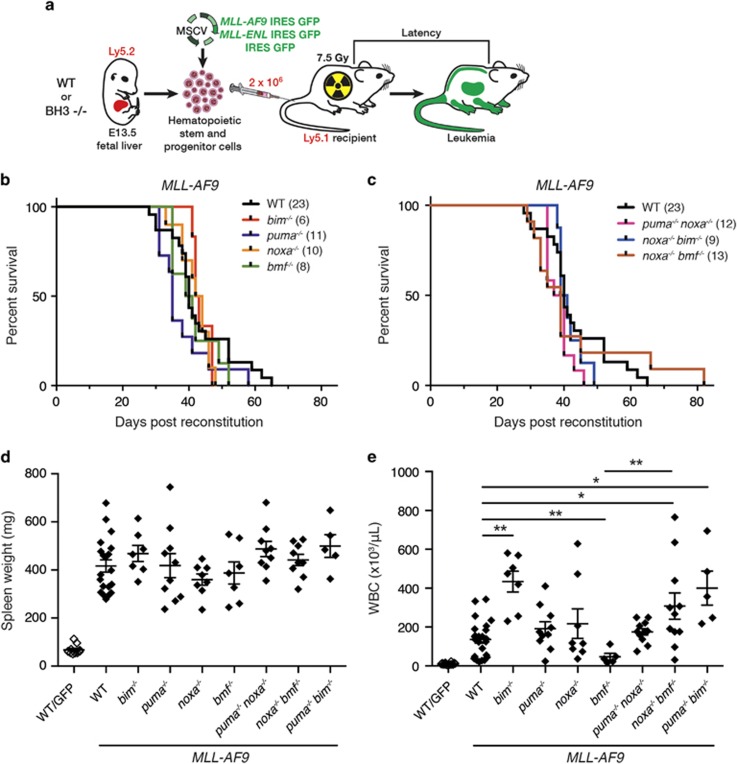
Generation of murine AMLs. (**a**) E13.5 fetal liver cells from WT or BH3-only gene KO Ly5.2 C57BL/6 mice were infected with *MLL-AF9*/GFP, *MLL-ENL*/GFP or GFP MSCV retrovirus (Materials and methods section) and injected into sub-lethally irradiated (7.5 Gy) C57BL/6-Ly5.1 mice (2 × 10^6^ cells/mouse). (**b**,**c**) Kaplan–Meier survival analysis of mice transplanted with fetal liver cells of the indicated genotype after infection with *MLL-AF9* virus; number of recipient mice is indicated in brackets. Mice were monitored regularly and euthanized humanely when showing signs of AML-induced stress. (**d**) Spleen weight and (**e**) WBC count of individual mice at autopsy. Data points represent individual mice with mean and S.E.M. indicated. *P-*values were determined by unpaired *t* test with Welsh's correction for differences in variance. *P<0.05, **P<0.01. Comparable data for mice transplanted with *MLL-ENL* virus-exposed cells is shown in Supplementary Figure S1

*MLL-AF9* and *MLL-ENL* driven AMLs were generated using retroviral transduction of fetal liver cells from wild type (WT) or BH3-only gene knock out (KO) mice and hematopoietic reconstitution of sub-lethally irradiated mice,^39, 40, 41^ as described in Figure 1a. The reconstituted mice are designated hereafter according to the virus and the genotype of the fetal liver cells (e.g., WT/green fluorescent protein (GFP) indicates mice reconstituted with WT fetal liver cells exposed to control GFP virus and *noxa*^−/−^*/MLL-AF9* indicates mice reconstituted with *noxa*^−/−^ fetal liver cells exposed to *MLL-AF9* GFP virus).

Irrespective of the genotype of the donor stem/progenitor cells, AML developed in all mice reconstituted with *MLL-AF9* or *MLL-ENL* virus-infected cells, most needing to be euthanized within a period of 30–65 days (Figures 1b and c and Supplementary Figure S1a). The sick mice had an enlarged spleen and elevated blood leukocytes (Figures 1d and e,Supplementary Figures S1b and c and Supplementary Table S1), as well as thrombocytopenia and anemia (Supplementary Figure S2). In contrast, GFP mice of all genotypes remained healthy until culled, usually after 120 days.

Loss of Bim, Puma, Noxa, Bmf or combinations thereof, made no significant difference to the kinetics of morbidity (Figures 1b and c and Supplementary Figure S1a) or degree of splenomegaly (Figure 1d,Supplementary Figure S1b), although circulating leukocytes were significantly elevated in sick *bim*^−/−^/*MLL-AF9* and *bim*^−/−^/*MLL-ENL* mice compared with their WT/*MLL-AF9* and WT/*MLL-ENL* counterparts, principally due to a greater increase in myeloid cells (Mac1^+^Gr1^+^; see Supplementary Table S1). Curiously, blood leukocytes were not as elevated in sick *bmf* ^−/−^/*MLL-AF9* mice as in sick WT *MLL-AF9* mice but were comparable in *bmf*^−/−^/*MLL-ENL* mice (Figure 1e and Supplementary Figure S1c).

Sick mice were autopsied and tissues subjected to histological and flow cytometric analysis (Supplementary Figure S3,Supplementary Table S1 and data not shown). As reported by others,^39, 40, 41^ the bone marrow and blood were replete with differentiated myeloid (Mac1^+^/Gr1^+/−^) cells. Normal splenic architecture was effaced by these abundant malignant myeloid cells, which also infiltrated the liver, kidney and other tissues. WT and BH3-only gene KO *MLL-AF9* and *MLL-ENL* AMLs had comparable pathology (data not shown).

### Expression of apoptosis regulators

**Figure 2:**
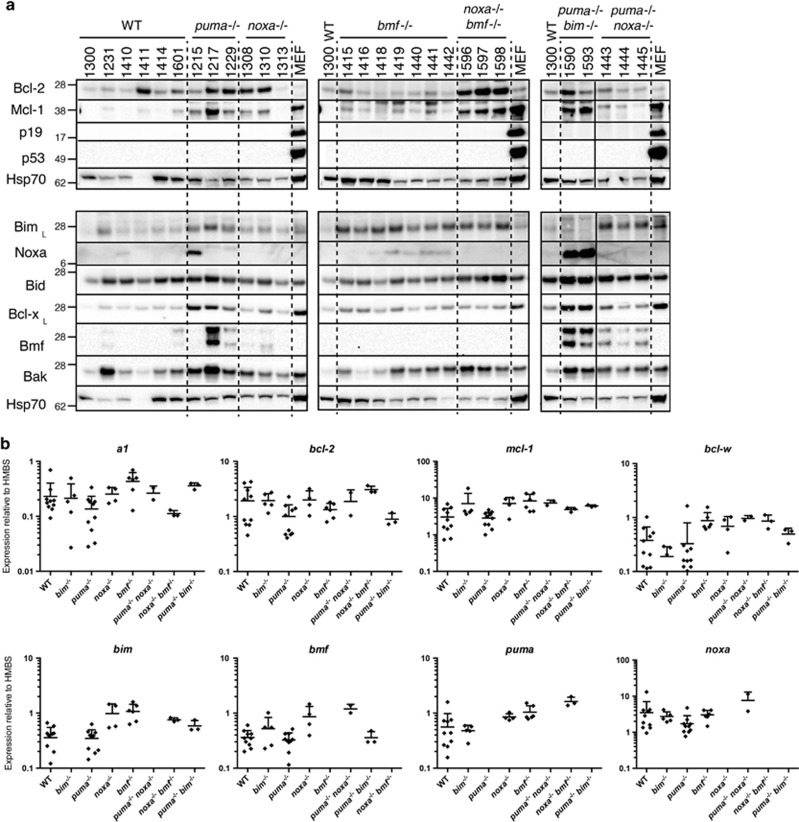
Expression of apoptosis regulatory genes in *MLL-AF9* AMLs. (**a**) Western blot and (**b**) qPCR analysis of expression of p53, p19Arf and *bcl-2* gene family members in bone marrow cells of sick primary *MLL-AF9* AML mice (Supplementary Methods). Protein and RNA were prepared from bone marrow taken at autopsy. Panels in **a** indicate six separate gels, each of which included WT AML #1300, with dotted lines separating different genotypes (the solid line in panel 3 indicates electronic removal of a lane). SV40 transformed mouse embryonic fibroblasts (MEF) served as positive controls for p53 and p19Arf expression; Hsp70 served as a loading control. Molecular weight (kD) markers are indicated. The following AMLs were analyzed by qPCR: WT #1223, 1224, 1155, 1156, 1231, 1232, 1601, 1410, 1411, 1414; *bim*^−/−^ #1249, 1250, 1213, 1157, 1158; *puma*^−/−^ #1230, 1214, 1215, 1216, 1217, 1218, 1225, 1226, 1228, 1229; *noxa*^−/−^ #1259, 1313, 1308, 1310; *bmf*^−/−^ #1440, 1441, 1442, 1416, 1419, 1418; *puma*^−/−^ *bim*^−/−^ # 1590, 1593; *noxa*^−/−^ *bmf* ^−/−^ #1596; 1597; 1598; *puma*^−/−^ *noxa*^−/−^ #1443; 1444, 1445. qPCR analysis was relative to that of hydroxymethylbilane synthase (HMBS). Error bars indicate S.D. Data for additional Bcl-2 family members is provided in Supplementary Figure S4

To further characterize the *MLL-AF9* AMLs, we ascertained the expression pattern of key apoptosis regulators by western blot and quantitative PCR analysis of bone marrow cells from sick primary mice (4–10 per genotype; Figure 2 and Supplementary Figure S4). In addition, western blots were performed on spleen cells (Supplementary Figure S5).

Pro-survival Bcl-2 family proteins Bcl-2, Bcl-x_L_, Mcl-1 and A1 were detected in all AML genotypes analyzed at variable levels. No suitable antibody was available for Bcl-w protein but *bcl-w* transcript levels were comparable in the WT, *bim*^−/−^ and *puma*^−/−^ *MLL-AF9* AMLs and somewhat higher in AMLs of other genotypes.

Pro-apoptotic Bax and Bak proteins were readily detected in all AMLs examined. BH3-only proteins Bim, Puma, Bmf and Bad were apparent in most and Bid was seen in all (except those lacking the corresponding gene). Noxa protein was detectable in some *bim*^−/−^, *puma*^−/−^, *bmf* ^−/−^ and *puma*^−/−^*bim*^−/−^ AMLs but not in any of the WT AMLs, although *noxa* transcripts were detectable in the WT AMLs.

In general, the AMLs appeared to have higher expression of Bcl-2 family members than Mac1^+^Gr1^+^ cells isolated by flow cytometry from normal bone marrow (not shown).

Expression of p53 or p19Arf protein in the absence of an apoptotic stimulus is indicative of mutation or loss of p53 respectively.^44^ However, none of 27 AMLs tested expressed detectable levels of either of these proteins (Figure 2a), suggesting that mutations affecting the p53 pathway were rare, as is also the situation in human AML.^2^

### Drug sensitivity of WT and BH3-only gene KO AMLs

To ascertain the *in vitro* drug sensitivity of the AMLs, short-term cell lines were established from multiple primary tumors of each genotype by culturing bone marrow or spleen cells from sick mice (Materials and methods section). The drugs tested included the genotoxic agents cytarabine (cytosine arabinoside), daunorubicin and etoposide as well as ABT-737 and the proteasome inhibitor bortezomib. Drug doses were guided by published data^2, 45, 46^ and preliminary dose response tests.

**Figure 3:**
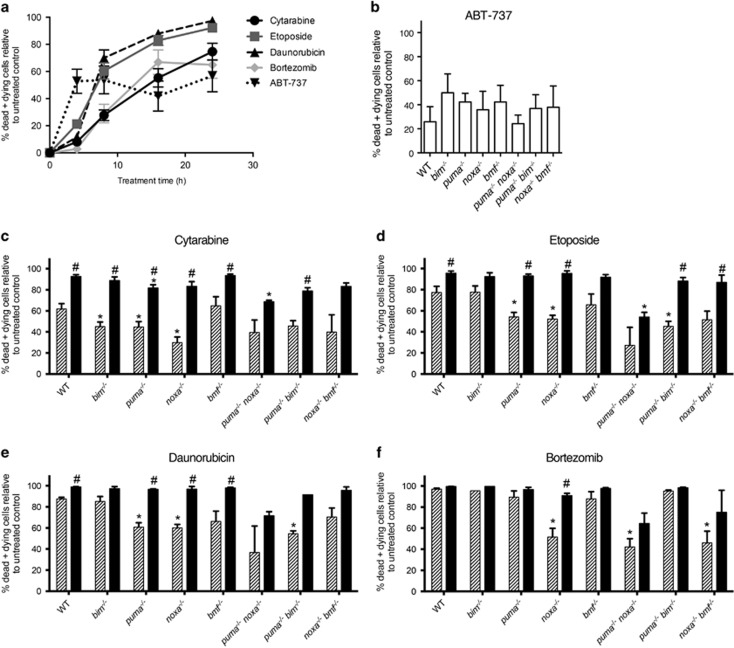
*In vitro* sensitivity of *MLL-AF9* AML cell lines to cytotoxic agents. Drug sensitivity tests were performed using short-term cell lines established from bone marrow of sick primary *MLL-AF9* AML mice (Materials and methods section). (**a**) WT/*MLL-AF9* AMLs (*n*=4) were treated with standard chemotherapeutic drugs cytarabine (300 ng/ml), etoposide (300 ng/ml), daunorubicin (50 ng/ml), bortezomib (5 nM) or BH3 mimetic ABT-737 (1 *μ*g/ml), harvested at 4, 8, 16 and 24 h and analyzed by flow cytometry following staining with annexin V-Alexa Fluor 647 and propidium iodide (dying and dead cells are positive for annexin V or both markers). Results are expressed as percentage of dead and dying cells relative to that of cells cultured in parallel in medium alone. Value shown is mean±S.E.M. (**b–f**) AMLs of the indicated genotypes were cultured for 16 h in the presence of ABT-737 alone (**b**); or with cytarabine (**c**), etoposide (**d**), daunorubicin (**e**) or the proteasome inhibitor bortezomib (**f**), each alone (gray) or in combination with ABT-737 (black). The percentage of dead and dying cells is expressed relative to cells of the same genotype incubated in the absence of drug(s). Values represent mean±S.E.M. Data plotted for ABT-737 alone were pooled from all experiments (*n*=8 independent lines for WT and *n*=2–6 independent lines for other genotypes). The number of independent lines used for other drugs±ABT-737 were: WT *n*=5; *bim*^−/−^ *n*=3, except with bortezomib where *n*=1; *puma*^−/−^ *n*=3; *noxa*^−/−^ *n*=3; *bmf*^−/−^ *n*=4; *puma*^−/−^*noxa*^−/−^ *n*=2; *puma*^−/−^*bim*^−/−^ *n*=2; *noxa*^−/−^*bmf*^−/−^ *n*=3. *P-*values were calculated using an unpaired *t* test with Welch's correction. *P<0.05 between WT AMLs and the indicated BH3-only gene KO AMLs in response to a particular drug or combination; ^#^*P*<0.05 for drug in combination with ABT-737 versus just the single drug alone for the same genotype

Figure 3a shows the kinetics of cell death obtained for WT/*MLL-AF9* AMLs (*n*=4) exposed to each of these drugs. Killing with ABT-737 was more rapid than with the other agents, particularly cytarabine, but then plateaued. For subsequent studies, a 16 h time point was chosen and the cytarabine concentration was increased to 600 ng/ml to increase its efficacy.

To assess which BH3-only proteins might be critical for individual drug regimens, we compared the viability of the various BH3-only gene KO/*MLL-AF9* AMLs with those of WT/*MLL-AF9* AMLs treated in parallel. Three or more independent lines were tested for each genotype, except the *puma*^−/−^*noxa*^−/−^ and *puma*^−/−^*bim*^−/−^ lines for which only two were available. Figure 3b shows the results for treatment with ABT-737 alone and Figure 3c–f for treatment with cytarabine, etoposide, daunorubicin or bortezomib, either alone (gray) or in combination with ABT-737 (black). An asterisk above a column indicates significantly greater resistance for specific BH3-only gene KO AMLs than WT AMLs and a hash indicates significantly better response of the indicated genotype to the combination therapy than to the single drug.

Neither Bim, Puma, Noxa or Bmf was essential for ABT-737 cytotoxic activity, since none of the corresponding gene KO AMLs displayed significant resistance to this BH3 mimetic, and neither did any of the double BH3-only gene KO AML lines tested (Figure 3b).

Loss of Puma and/or Noxa increased resistance to the DNA damaging agents cytarabine, etoposide and daunorubicin. Loss of Bim increased resistance to cytarabine but not etoposide or daunorubicin and loss of Bmf had no significant impact for any of the drugs (Figures 3c–e).

Encouragingly, WT AMLs were highly sensitive to treatment with bortezomib, as were *bim*^−/−^, *puma*^−/−^ and *bmf*^−/−^ AMLs (Figure 3f). Only loss of Noxa conferred significant resistance to bortezomib, suggesting that, for this type of leukemia, Noxa is the primary trigger for apoptosis mediated via bortezomib.

ABT-737 improved the response of WT/*MLL-AF9* AMLs to cytarabine, etoposide and daunorubicin (*P*<0.05). It also enhanced the sensitivity of *puma*^−/−^, *noxa*^−/−^ and *bmf*^−/−^ AMLs to each of these drugs and of *bim*^−/−^ AMLs to cytarabine. Furthermore, despite their resistance to bortezomib as a single agent, *noxa*^−/−^/*MLL-AF9* AMLs appeared sensitive to the combination.

**Figure 4:**
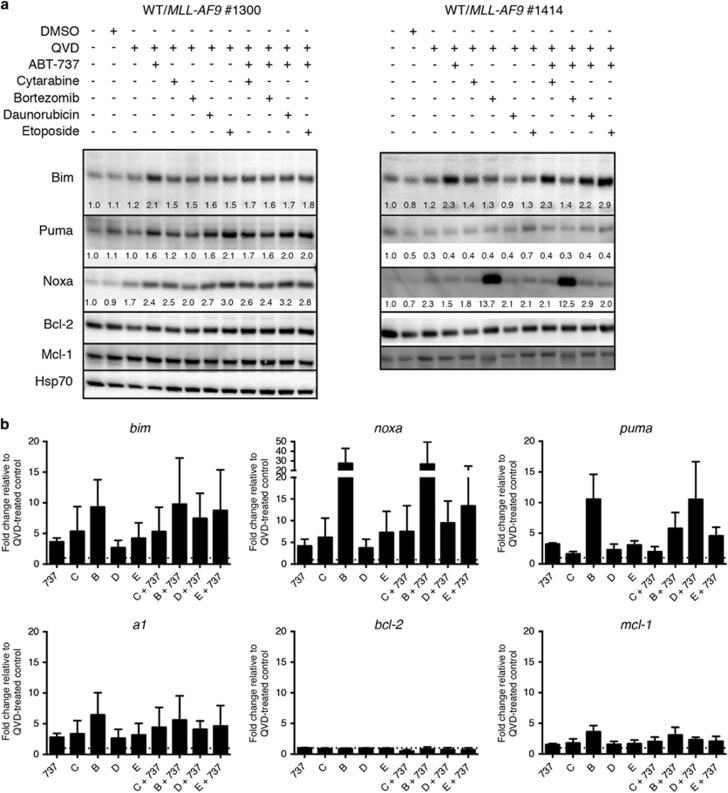
Expression of Bcl-2 family members following treatment. Expression of Bcl-2 family members in cultured primary WT/*MLL-AF9* AMLs following treatment with cytotoxic agents. (**a**) Western blot analysis of protein expression in AMLs #1300 and #1414 after 6 h treatment with the indicated drugs. Numbers below bands indicate the intensity of each lane relative to the untreated control for each antibody. Quantitation was performed using Image Lab software. (**b**) qPCR analysis of RNA expression in AMLs #1300, #1411 and #1601 after 3 h treatment. ΔΔCT values normalized to HMBS control and made relative to cells treated with QVD only to determine drug-induced fold change. Data represents mean fold change±S.E.M., with dashed line indicating a value of 1; note the different axis for *noxa* expression. Comparable PCR analyzes of BH3-only gene KO/*MLL-AF9* AMLs are summarized in Supplementary Figure S7. AML cell lines maintained in culture in IMDM with 10% FCS and supplemental IL-3 (Materials and methods section) were treated with 600 ng/ml cytarabine [C], 25 nM bortezomib [B], 200 ng/ml daunorubicin [D], 300 ng/ml etoposide [E], either alone or in combination with 1 *μ*g/ml ABT-737 [737] as indicated, in presence of the pan-caspase inhibitor QVD-OPH (25 *μ*M)

In an effort to better understand the responses triggered by the various drugs, three WT/*MLL-AF9* cell lines (#1300, #1414 and #1411) and one *bmf* ^−/−^/*MLL-AF9* line (#1415) were treated in the presence of the pan-caspase inhibitor QVD-OPH and analyzed after 6 h by western blot (Figure 4a and Supplementary Figure S6). Exposure to bortezomib markedly increased the levels of Noxa protein in two lines (#1414 and #1415) but not the others. Puma levels modestly increased in response to etoposide and Bim increased with ABT-737. Bcl-2 and Mcl-1 protein levels remained relatively constant in the face of all agents.

Comparable qPCR studies were performed 3 h after treatment for three WT/*MLL-AF9* (Figure 4b) and various BH3-only gene KO/*MLL-AF9* lines (Supplementary Figure S7 and data not shown). *Noxa* transcripts increased markedly in the WT AMLs following bortezomib treatment (Figure 4b) and also in the *bim*^−/−^ AMLs (Supplementary Figure S7) but only modestly in the *puma*^−/−^ AMLs. *Puma* RNA was elevated in WT AMLs in response to bortezomib (Figure 4b) and in Bim-deficient AMLs in response to most agents, particularly cytarabine, bortezomib and the various drug combinations. Smaller, more variable increases in *bim, a1* and *mcl-1* RNAs were noted in response to most agents but there was no significant change in *bad*, *bid*, *bmf*, *bcl-2*, *bcl-w*, *bcl-x*_*L*_RNAs, for either WT or BH3-only gene KO AMLs.

### *In vivo* treatment of *MLL-AF9* AMLs

**Figure 5:**
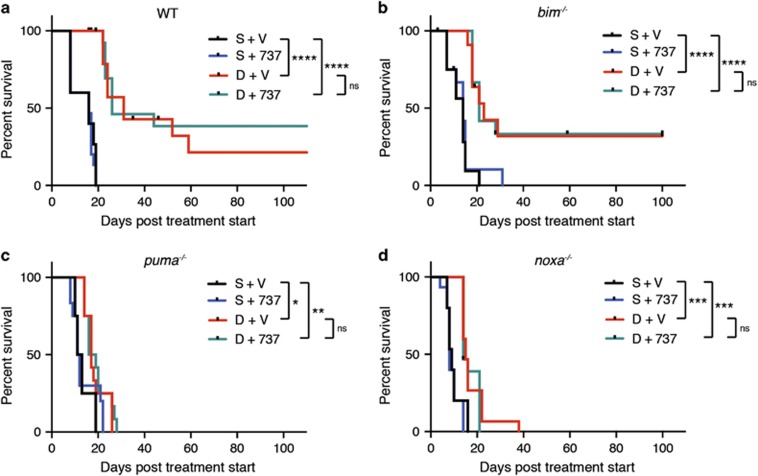
*In vivo* treatment of *MLL-AF9* AMLs with daunorubicin in combination with ABT-737. Kaplan–Meier survival curves for mice transplanted with *MLL-AF9* AMLs. 8–10-week-old Ly5.1 C57BL/6 mice (non-irradiated) were injected with 0.5 × 10^6^ bone marrow cells from sick secondary WT/*MLL-AF9* or BH3-only gene KO/*MLL-AF9* mice (three recipients per tumor per treatment arm) and treatment was started 3 days later: 5 mg/kg daunorubicin [D] intravenously on days 1, 4 and 9 and/or 75 mg/kg ABT-737 [737] intraperitoneally on days 1–5 and 8–12; controls received saline [S] and ABT-737 vehicle [V]. Transplanted mice were monitored daily for symptoms of AML and euthanized if morbidly ill or at the end of experiment (100 days post treatment start). A total of (**a**) 5 WT/*MLL-AF9* AMLs (#1211, 1223, 1224, 1411 and 1414), (**b**) 4 *bim*^−/−^ *MLL-AF9* AMLs (#1158, 1213, 1249 and 1250), (**c**) 4 *puma*^−/−^ *MLL-AF9* AMLs (#1218, 1225, 1226 and 1229) and (**d**) 5 *noxa*^−/−^ *MLL-AF9* AMLs (#1306,1308, 1309, 1310 and 1311) were tested (see also Supplementary Figure S8 for individual tumor results). Statistical significance was determined by Log-rank (Mantle-Cox) test. *P<0.05, **P<0.01, ***P<0.001, ****P<0.0001. Daunorubicin treatment alone, or in combination with ABT-737, significantly prolonged survival of WT/*MLL-AF9* and *bim*^−/−^/*MLL-AF9* AMLs compared with saline treatment (*P*<0.0001), *puma*^−/−^/*MLL-AF9* had significantly prolonged survival when treated with daunorubicin alone (*P*=0.0173) or with daunorubicin in combination with ABT-737 (*P*=0.0026), as did *noxa*^−/−^/*MLL-AF9* when treated with daunorubicin alone (*P*=0.0006) or in combination with ABT-737 (*P*=0.0001). For all genotypes there was no significant difference (*P*>0.5) in survival between daunorubicin treatment and treatment with the combination of daunorubicin and ABT-737

We next embarked on *in vivo* trials comparing the efficacy of traditional and novel therapeutic regimens for treating AML-bearing mice. Figure 5 presents results obtained for daunorubicin, either alone or in combination with ABT-737 (see Supplementary Figure S8 for results obtained for individual AMLs). Healthy mice were injected with 0.5 × 10^6^ bone marrow cells from sick secondary *MLL-AF9* mice (3 recipients per AML per treatment arm) and treatment was started 3 days later. Mice received 5 mg/kg body weight daunorubicin intravenously on days 1, 4 and 9 and either ABT-737 (75 mg/kg body weight) or vehicle intraperitoneally on days 1–5 and 8–12.

For the WT/*MLL-AF9* AMLs (Figure 5a), controls receiving saline and vehicle (black) all died within 20 days and those treated with ABT-737 alone (blue) fared no better. In contrast, daunorubicin (red) significantly extended lifespan; of the 15 mice transplanted with five different WT/*MLL-AF9* AMLs, almost 50% survived for more than 30 days, and two were still alive at the end of the experiment (100 days; Figure 5a).

Treatment with ABT-737 as well as daunorubicin did not significantly increase survival over daunorubicin alone (compare aqua and red lines), although more mice achieved long-term survival (5/15 versus 2/15). No overt correlation was evident between the degree of responsiveness of individual AMLs (Supplementary Figure S8) and their pattern of expression of pro-survival Bcl-2 proteins or BH3-only proteins (Figure 2 and Supplementary Figure S5). RNASeq analysis is being undertaken to attempt to account for the differences in responsiveness.

Of note, while *MLL-AF9* AMLs lacking Bim (Figure 5b) responded similarly to WT/*MLL-AF9* AMLs, those lacking either Puma (Figure 5c) or Noxa (Figure 5d), were very resistant to daunorubicin, even when it was combined with ABT-737. Thus, Puma and Noxa are critical for the cytotoxic action of daunorubicin.

**Figure 6:**
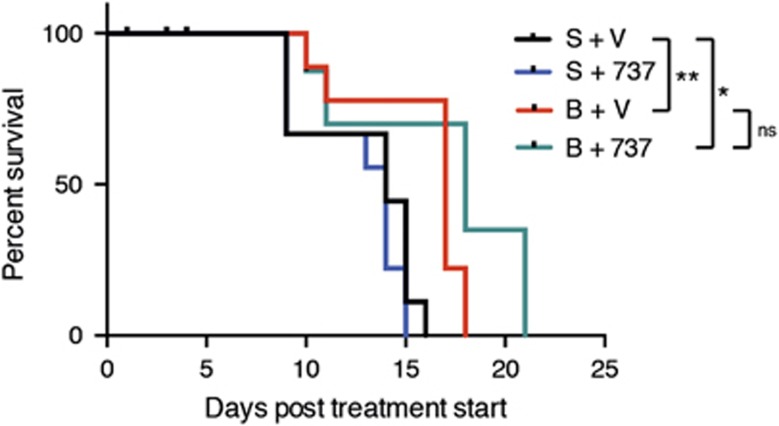
*In vivo* treatment of WT/*MLL-AF9* AMLs with bortezomib in combination with ABT-737. Kaplan–Meier survival curves for mice transplanted with WT/*MLL-AF9* AMLs and treated with bortezomib with or without ABT-737. 8–10-week-old Ly5.2 C57BL/6 mice (non-irradiated) were injected with 0.5 × 10^6^ bone marrow cells from sick secondary WT/*MLL-AF9* mice (3–6 recipients per tumor per treatment arm) and treatment was started 3 days later: 0.75 mg/kg bortezomib [B] or saline [S] intravenously on days 1, 4, 8 and 11 of treatment and/or 75 mg/kg ABT-737 [737] or vehicle [V] intraperitoneally on days 1–5 and 8–12 of treatment. Transplanted mice were monitored daily and euthanized if morbidly ill, either from bortezomib toxicity within the first week (censored) or from typical AML. A total of 3 WT/*MLL-AF9* AMLs (#1224, 1223 and 1414) were tested. Statistical significance was determined by Log-rank (Mantel–Cox) test. Treatment with bortezomib versus vehicle significantly prolonged survival (*P*=0.002), as did treatment with bortezomib plus ABT-737 (*P*=0.019). There was no significant difference between treatment with bortezomib alone compared with the combination of bortezomib and ABT-737 (*P*=0.116)

In view of the high sensitivity of the AML lines to bortezomib *in vitro*, we were keen to test its efficacy *in vivo*. Unfortunately, tests performed on healthy C57BL/6 mice indicated relatively poor tolerance of the drug, evidenced by severe weight loss. AML-bearing mice were given the maximum tolerated dose (0.75 mg/kg) and any mice euthanized early due to weight loss (usually before 7 days) were censored. Figure 6 summarizes the outcome for mice developing AML following transplantation with three different WT/*MLL-AF9* AMLs. Treatment with bortezomib provided a significant extension of life although, at this dose, efficacy was not as great as with daunorubicin, and combination therapy with ABT-737 provided no additional benefit.

## Discussion

In this study, we tested the impact of loss of individual BH3-only proteins on the development and treatment of AMLs driven by MLL-fusion genes. The AMLs were generated by transplanting WT or BH3-only gene KO fetal liver cells infected with *MLL-AF9* or *MLL-ENL* virus into sub-lethally irradiated recipient mice. Of note, loss of Bim, Puma, Noxa, Bmf, or combinations thereof, made no significant difference to the kinetics of morbidity (Figure 1 and Supplementary Figure S1), all recipients developing florid AML within 30–65 days. These results differ markedly from those obtained for lymphomagenesis in E*μ*-*myc* mice, where disease was accelerated by loss of Bim, Puma or Bmf, although not Noxa.^47, 48, 49^ Thus, in contrast to Myc-driven lymphomagenesis, none of the BH3-only proteins tested appear to serve as a critical tumor suppressor for the development of MLL-driven AML. This is suggestive of redundant roles. However, the faster morbidity of the retroviral AML model (median 6 weeks) compared with the E*μ*-*myc* lymphoma model (median 15 weeks) may have masked a tumor suppressor role.

To ascertain the sensitivity of the MLL fusion protein-driven AMLs to conventional and targeted cytotoxics, we first tested primary cell lines established by culturing bone marrow cells from sick reconstituted mice in medium supplemented with IL-3. Each of the standard chemotherapeutic drugs was relatively effective at killing WT/*MLL-AF9* AMLs, etoposide and daunorubicin more so than cytarabine at the concentrations tested. As might be predicted for DNA damaging agents such as these, loss of either Puma or Noxa, both p53 targets, increased resistance. Loss of Bim increased resistance to cytarabine but not to etoposide or daunorubicin, whereas loss of Bmf had no significant impact. ABT-737 was not very effective as a single agent but significantly improved the response to the standard agents suggesting that it may improve clinical response or allow lower concentrations of traditional cytotoxics to be used.

Bortezomib was highly effective at killing WT/MLL-AF9 AMLs *in vitro*. Significantly, whereas *puma*^−/−^, *bim*^−/−^ and *bmf*^−/−^ AMLs were as sensitive as WT/*MLL-AF9* AMLs, Noxa-deficient AMLs displayed increased resistance. Noxa RNA levels increased markedly within 3 h in primary WT/*MLL-AF9* lines exposed to bortezomib and high levels of Noxa protein were observed within 6 h in two bortezomib-treated lines (one WT and the other *bmf*^−/−^) but not in two other WT lines that were nevertheless highly sensitive to bortezomib (Figure 4a and Supplementary Figure S6). Differential kinetics of Noxa induction or breakdown may explain the varied results.

Several mechanisms have been implicated in the cytotoxicity induced by proteasome inhibitors, including stabilization of p53 and I*κ*B, and induction of ER stress and the unfolded protein response (reviewed in refs 50, 51), all of which trigger apoptosis through the Bcl-2 family-regulated pathway. The apparent lack of Puma-dependence implies that Noxa induction by bortezomib in *MLL-AF9* AML cells occurs through a p53-independent mechanism. Noxa transcription can be upregulated by *c-myc* in tumor cells in response to bortezomib.^52^

Noxa strongly binds to and inhibits anti-apoptotic Mcl-1,^20, 21^ thereby facilitating activation of Bak and Bax. Since Noxa promotes Mcl-1 degradation via the BH3-only E3 ubiquitin ligase Mule,^53^ Mcl-1 levels might be expected to rise in bortezomib-treated cells but this was not apparent for the AML lines (Figure 4a). Presumably bortezomib-mediated Noxa up-regulation titrates the level of Mcl-1 sufficiently to kill the cells.

We transplanted five independent WT/ *MLL-AF9* AMLs into immunocompetent mice to test their sensitivity *in vivo* to daunorubicin and bortezomib, both alone or in combination with ABT-737. Daunorubicin treatment significantly prolonged the life of most AML-bearing mice, some of which (7/30) survived until the end of experiment (100 days post treatment; Figure 5). Although the addition of ABT-737 did not further improve survival significantly, given the *in vitro* results discussed above it would be worthwhile testing whether combination therapy enabled a lower dose of daunorubicin to be effective *in vivo*.

Importantly, our data clearly show, for the first time, that effective killing of *MLL-AF9* AML cells by daunorubicin *in vivo* is dependent on expression of Puma and Noxa, as the AMLs lacking these BH3-only genes were more refractory to treatment (compare Figures 5c and d with a). Bim appears to play no role (Figure 5b), in contrast to the situation observed for E*μ*-*myc* lymphomas where Bim, Puma and Noxa were all involved in killing by DNA damage-inducing drugs.^54^ Although not as efficacious as daunorubicin, bortezomib also extended the life of AML-transplanted mice (Figure 6) and the *in vitro* data suggests this response is highly Noxa-dependent.

In summary, our mouse genetic studies have revealed the importance of the BH3-only proteins Puma and Noxa for the efficacy of cytotoxic drugs currently used to treat *MLL-AF9* AML. Our tests with the BH3 mimetic ABT-737, which is specific for Bcl-2, Bcl-x_L_ and Bcl-w,^28, 29^ suggest that its orally available derivative, ABT-263 (ref. 30) or Bcl-2-specific ABT-199,^31^ may prove beneficial in combination therapy with either genotoxic or other drugs. However, given the widespread and robust expression of Mcl-1 and A1 in AMLs, their dependence on Mcl-1 (ref. 41) and the susceptibility of *MLL-AF9* AMLs to Noxa-induced killing (this paper), Noxa-like BH3 mimetics currently being developed to target Mcl-1 (and/or A1/BFL1) may prove more efficacious.

## Materials and Methods

### Mice

The *noxa*^−/−^,^55^ *puma*^−/−^,^55^ *bim*^−/−^ (ref. 56) and *bmf*^−/−^ (ref. 57) mice have been described previously; all were maintained on a C57BL/6 J (Ly5.2) background at the Walter and Eliza Hall Institute (WEHI). C57BL/6-Ly5.1 mice were originally obtained from Jackson Laboratories. Experimental protocols were approved by WEHI's Animal Ethics Committee.

### AML generation

MSCV retroviruses encoding internal ribosomal entry site (IRES)/GFP, *MLL-AF9*/IRES/GFP^39^ or *MLL-ENL*/IRES/GFP DNA^58^ were prepared by transfection of Phoenix cells.^59^ For infection, cells prepared from three cryopreserved fetal livers of each genotype in stem cell medium (SCM; Iscove's modified Dulbecco's medium (IMDM) containing 20% fetal bovine serum, 100 ng/ml stem cell factor, 50 ng/ml thrombopoietin and 50 ng/ml Flt-3 ligand, 10 ng/ml IL-6; all obtained from WEHI cytokine facility), were incubated at 37 °C in 10% CO_2_ for 24 h, plated at 1 × 10^6^ viable cells/ml into 12-well plates coated with retronectin containing an equal volume (1 ml) of fresh virus-containing supernatant in SCM and then polybrene was added to 4 *μ*g/ml. Following centrifugation at 2500 r.p.m. (1360*g*) for 1 h at 32 °C, the cells were incubated overnight at 37 °C. A second ‘spin infection' was performed the next day, using fresh virus. After incubation overnight, cells were detached using a rubber policeman, washed in PBS, resuspended at 10^7^ viable cells/ml then injected into the tail vein of sub-lethally irradiated (7.5 Gy) Ly5.1 mice (200 *μ*l/mouse). Mice were monitored regularly for AML symptoms: hunched stance, ruffled coat, lethargy, anemia and splenomegaly. Mouse survival analysis utilized GraphPad Prism and significance was determined using Log-rank (Mantel–Cox) test.

### *In vitro* drug treatment

Bone marrow-derived primary AML cells were cultured in IMDM supplemented with 10% fetal bovine serum (FBS) and IL-3-containing culture supernatant (WEHI). Aliquots of 100 *μ*l were plated into 96-well plates (1 × 10^5^ cells/well) and incubated for 16 h with 600 ng/ml cytarabine, 300 ng/ml etoposide, 50 ng/ml daunorubicin or 5 nM bortezomib, either alone or in combination with 1 *μ*g/ml ABT-737. Following treatment, cells were washed once with balanced salt solution (150 mM NaCl, 3.7 mM KCl, 2.5 mM CaCl_2_, 1.2 mM MgSO_4_, 7.4 mM HEPES.NaOH, 1.2 mM KH_2_PO_4_ and 0.8 mM K_2_HPO_4_) containing 5% FBS and resuspended in the same medium with Annexin V-Alexa Fluor 647 (kindly provided by Daniel Gray, WEHI) and 4 *μ*g/ml propidium iodide. Cell viability was determined on an LSR I flow cytometer using FlowJo software.

### *In vivo* drug treatment

Primary tumors (T0) were expanded by transplanting 2 × 10^6^ spleen cells via tail vein injection into C57BL/6 Ly5.1 mice. Bone marrow cells of sick recipients (T1) were cryopreserved. For treatment studies, non-irradiated mice (8–10 week female C57BL/6 Ly5.1) were injected intravenously with 0.5 × 10^6^ bone marrow-derived T1 tumor cells and drug regimens commenced 3 days later: on days 1, 4 and 9, 5 mg/kg body weight daunorubicin or an equal volume (100 *μ*l) of saline was injected intravenously followed by a flush of saline (700 *μ*l) using a butterfly catheter; on days 1, 4, 8 and 11, 0.75 mg/kg body weight bortezomib or an equal volume (100 *μ*l) of saline was injected intravenously; on days 1–5 and 8–12, 75 mg/kg body weight ABT-737 (dissolved in 10% dimethyl sulfoxide and 90% vehicle and titrated to pH 4.0 using 1 M Hepes) or an equal volume of vehicle (65% glucose-anhydrous BP 1.0, 30% propylene glycol, 5% Tween 80)^33^ was injected intraperitoneally. The daunorubicin and bortezomib doses were based on initial dose escalation toxicity tests in healthy C57BL/6 mice; tail necrosis, the limiting toxicity for daunorubicin, was subsequently overcome by flushing with saline; the ABT-737 dose was based on published data.^60^ Mice were euthanized when showing severe AML symptoms, significant weight loss (>15% of initial body weight), or after the experimental end point (100 days).

### Molecular analysis

Procedures used for immunoblotting and RT-PCR are provided in the Supplementary Methods.

## Abbreviations

BH3: Bcl-2 homology domain 3
MLL: Mixed lineage leukemia
MLL-ENL: Fusion gene combining MLL with MLLT1
MLL-AF9: Fusion gene combining MLL with MLLT3
AML: Acute myeloid leukemia
KO: Knock out
WT: Wild type
GFP: Green fluorescent protein
IRES: Internal ribosomal entry site
SCM: Stem cell medium
IMDM: Iscove's modified Dulbecco's medium
FBS: Fetal bovine serum
ROS: reactive oxygen species
