# Supplementary material for: Impact of loss of BH3-only proteins on the development and treatment of MLL-fusion gene-driven AML in mice
Source: Cell Death Dis. 2016 Sep 1;7(9):e2351–. doi: 10.1038/cddis.2016.258 (PMC5059861; doi:10.1038/cddis.2016.258)
Supplement: Supplementary Information [file cddis2016258x1.pdf]

Supplemental Data

Methods

Analysis of mice with AML

Moribund mice were euthanized and autopsied. Spleens were weighed and tissues collected for histology and immunophenotyping. Soft tissue and sternum were collected into Bouin's fixative or 10% formalin, respectively, before embedding in paraffin wax and staining with hematoxylin and eosin. Sections were assessed using an Olympus BX43 microscope and photographed using an Olympus DP72 camera. Blood smears were stained with May-Grunwald-Giemsa. Bone marrow cytospins were prepared using a Cytospin3 machine (Shandon) at 1000 rpm (113 x *g*) for 5 min with low acceleration. Peripheral blood was analyzed using an ADVIA 2120 hematology analyzer (Siemens, Erlangen, Germany). Cell composition of hematopoietic tissues was determined by flow cytometry: single cell suspensions were incubated with fluorescently conjugated antibodies and propidium iodide (PI) as previously described[61] and analyzed on an LSR I flow cytometer, gated for live single cells using FlowJo software (Tree Star, OR, USA).

Immunoblotting

Cell pellets were washed in cold PBS with 1 mM phenylmethanesulfonylfluoride (PMSF) and proteins extracted using RIPA buffer (150 mM NaCl, 5 mM EDTA, 50 mM Tris pH 8.0, 1 % Nonidet P40, 0.5 % sodium deoxycholate and 0.1 % SDS) containing a cocktail of protease inhibitors (Complete Protease Inhibitor cocktail, Roche) and 1 mM PMSF. Proteins (20 μg per lane) were resolved by SDS-PAGE electrophoresis using pre-cast 10% Bis-Tris NuPage gels (Thermo Fisher Scientific) and transferred to nitrocellulose using the iBlot system (Life Technologies). Blots were probed with antibodies listed below and visualized using Luminata™ Forte western HRP substrate (Merck-Millipore) on a ChemiDoc™ XRS+ Molecular Imager® (Bio-Rad). Antibodies used were: A1 (clone 6D7; WEHI mAb laboratory), β-actin (clone AC-74, Sigma #A2228), Bad (Enzo Life Sciences #IMG-5665), Bak (Sigma #B5987), Bax (clone 5B7; Sigma #B9054), Bcl-2 (clone 3F11; WEHI), Bcl-x_L (BD Biosciences #610212), Bid (clone 2D1-3; WEHI), Bim (clone 3C5; WEHI),

Bmf (clone 17C2; WEHI), HSP70 (clone N6; W Welch USCF), Mcl-1 (clone 19C4-15, WEHI), Noxa (ProSci #2437) and Puma (Abcam #ab27669).

RT-PCR

RNA was extracted from cells using Trizol (Thermo Fisher Scientific) and reverse transcribed into cDNA using the SuperScript III kit (Thermo Fisher Scientific). PCR was performed using TaqMan® primers (Life Technologies) and Taqman Gene Expression Master mix on an ABI7900 machine. Cycle threshold (CT) values were normalized to HMBS (hydroxymethylbilane synthase) cDNA. Taqman expression assays used were:

Bak Mm00432045_m1; Bax Mm00432050_m1;
Bad Mm00432042_m1; Bid Mm00432073_m1; Bim Mm00437796_m1;
Noxa Mm00451763_m1; Puma Mm00519268_m1; Bmf Mm00506773_m1;
Bcl-2 Mm00477631_m1; A1 Mm03646861_mH; Bcl-X_L Mm00437783_m1;
Bcl-w Mm03053297_s1; Mcl-1 Mm01257351_g1;
HMBS Mm01143545_m1

Table S1: Composition of haemopoietic tissues of primary reconstituted mice

		Wild type	*bim*$^{-/-}$	*puma*$^{-/-}$	*noxa*$^{-/-}$	*bmf*$^{-/-}$
**GFP**[1]						
Peripheral blood	WBC (x 10^6/mL)	11.8 ± 5.8 (7)	16.4 ± 14.6 (6)	7.82 ± 3.37 (4)		
	Mac1$^+$ Gr1$^-$	0.97 ± 1.0 (5)	0.66 ± 0.49 (5)	0.28 ± 0.25 (3)		
	Mac1$^+$ Gr1$^+$	0.62 ± 0.25 (5)	0.51 ± 0.33 (5)	0.21 ± 0.15 (3)*		
Spleen	Weight (mg)	67 ± 23 (6)	85 ± 46 (5)	48 ± 8 (5)		
	Total cells (x 10^7)	11.3 ± 4.4 (6)	13.9 ± 5.2 (5)	8.77 ± 2.57 (5)		
	Mac1$^+$ Gr1$^-$	0.53 ± 0.31 (5)	1.74 ± 3.03 (5)	0.43 ± 0.30 (5)		
	Mac1$^+$ Gr1$^+$	0.22 ± 0.10 (5)	0.16 ± 0.04 (5)	0.17 ± 0.06 (5)		
Bone marrow	Total cells (x 10^7)	4.24 ± 0.6 (6)	4.39 ± 0.62 (5)	4.33 ± 0.30 (5)		
	Mac1$^+$ Gr1$^-$	0.18 ± 0.05 (5)	0.53 ± 0.61 (5)	0.22 ± 0.10 (5)		
	Mac1$^+$ Gr1$^+$	1.49 ± 0.54 (5)	1.05 ± 0.15 (5)	1.59 ± 0.41 (5)		
MLL-AF9[1]						
Peripheral blood	WBC (x 10^6/mL)	147±79 (19)	422 ± 151 (6)**	186 ±102 (10)	233 ± 211 (9)	47.1 ± 38.7 (5)**
	Mac1$^+$ Gr1$^-$	58.3 ± 39.2 (6)	162 ±142 (4)	96.2 ± 71.5 (9)	121 ± 116 (5)	
	Mac1$^+$ Gr1$^+$	38.2 ±24.8 (6)	216 ±103 (4)*	47.3 ± 30.4 (9)	120 ± 93 (5)	
Spleen	Weight (mg)	429 ± 119 (13)	477 ± 95 (6)	418 ± 158 (10)	385 ± 47 (6)	412 ± 113 (6)
	Total cells (x 10^7)	37.2 ± 26.0 (4)		47.7 ± 15.0 (7)	71.3 ± 12.7 (6)	65.4 ± 33.4 (5)
	Mac1$^+$ Gr1$^-$	17.0 ± 15.4 (4)		23.0 ± 11.3 (7)	24.9 ± 7.0 (6)	21.8 ± 11.3 (5)
	Mac1$^+$ Gr1$^+$	11.7 ± 10.8 (4)		12.2 ±5.2 (7)	26.6 ± 7.7 (6)	29.2 ± 17.8 (5)
Bone marrow	Total cells (x 10^7)	6.26 ± 1.46 (3)		4.01 ± 1.04 (7)	6.68 ± 0.85 (6)	7.54 ± 2.33 (5)
	Mac1$^+$ Gr1$^-$	2.56 ± 0.74 (3)		2.49 ± 0.78 (7)	2.54 ± 1.01 (6)	3.00 ± 1.26 (5)
	Mac1$^+$ Gr1$^+$	2.14 ± 0.38 (3)		1.19 ± 0.31 (7)*	3.10 ± 0.83 (6)	3.59 ± 1.02 (5)
MLL-ENL[1]						
Peripheral blood	WBC (x10^6/mL)	227 ± 112 (4)	372 ± 138 (6)	207 ± 62 (6)	226 ± 98 (6)	179 ± 69 (5)
	Mac1$^+$ Gr1$^-$	105 ± 25 (4)	164 ± 74 (6)		57.7 ± 6.5 (2)*	54.4 ± 33.4 (3)
	Mac1$^+$ Gr1$^+$	92.8 ± 86.2 (4)	141 ± 93 (6)		50.0 ± 46.5 (2)	63.0 ± 19.6 (3)
Spleen	Weight (mg)	442 ± 125 (5)	550 ± 122 (6)	449 ± 94 (6)	453 ± 130 (2)	
	Total cells (x 10^7)	56.0 ± 16.8 (4)	68.9 ± 32.4 (6)	59.1 ± 8.6 (5)		
	Mac1$^+$ Gr1$^-$	27.1 ± 14.7 (4)	32.2 ± 18.1 (6)	20.6 ± 21.8 (2)		
	Mac1$^+$ Gr1$^+$	13.9 ± 6.4 (4)	19.2 ± 11.7 (6)	12.6 ± 2.8 (2)		
Bone marrow	Total cells (x 10^7)	3.74 ± 0.18 (4)	4.70 ± 1.11 (6)	4.48 ± 1.24 (5)		
	Mac1$^+$ Gr1$^-$	2.57 ± 0.82 (4)	2.48 ± 0.54 (6)	0.95 ± 1.17 (2)		
	Mac1$^+$ Gr1$^+$	0.94 ± 0.67 (4)	1.92 ± 1.11 (6)	0.91 ±1.42 (2)		

Values given are mean ± SD; the # of mice is indicated in brackets

[1]Virus used to infect fetal liver cells prior to transplantation into sub-lethally irradiated mice (see Methods)

p values were determined using t test with Welch's correction

*p <0.05 compared to WT

** p value <0.01 compared to WT

Figure S1: Generation of *MLL-ENL* AMLs. (**a**) Kaplan-Meier survival analysis of sub-lethally irradiated mice transplanted with fetal liver cells of the indicated genotype after infection with *MLL-ENL*/GFP retrovirus (see Materials and Methods); number of recipient mice is indicated in brackets. Mice were monitored regularly and sacrificed humanely when showing signs of AML-induced stress. (**b, c**) Spleen weight and WBC of individual mice at autopsy. P values were determined by unpaired t test with Welsh's correction for differences in variance. *p<0.05. Error bars indicate SEM.

Figure S2: **Abnormal platelet and hemoglobin levels in sick AML mice**. Comparison of platelet (**a, c**) and hemoglobin (**b, d**) levels in the blood of sick primary *MLL-AF9* (**a, b**) and *MLL-ENL* (**c, d**) AML mice and healthy control (WT/GFP) mice. Blood cell analysis was performed using an ADVIA 2120 hematology analyzer (Siemens). Data points represent individual mice with mean and SEM indicated. P values were determined by unpaired t test with Welsh's correction for differences in variance. *p<0.05

Figure S3: *MLL-AF9* AML phenotype in reconstituted mice. (**a**) Histology of bone marrow, spleen and liver of a typical sick WT/*MLL-AF9* mouse and a healthy control WT/GFP mouse. Bone marrow cytospins were stained with May-Grünwald Giemsa and spleen and liver sections were stained with hematoxylin and eosin. In WT/*MLL-AF9* mice, myeloid cells in the marrow are less differentiated than in WT/GFP mice and normal splenic architecture is effaced by infiltrating leukocytes, which are also prominent around blood vessels in the liver. (**b**) Flow cytometric

analysis of bone marrow. Mac^+Gr1^+ and $Mac1^+Gr1^-$ cells dominate *MLL-AF9* marrow compared to control marrow.

Figure S4: **Expression of Bcl-2 family members in *MLL-AF9* AMLs.** Gene expression was determined for primary WT/*MLL-AF9* and BH3-only gene KO/*MLL-AF9* AMLs by qPCR analysis of RNA prepared from bone marrow cells of primary transplanted mice. The results are expressed relative to HMBS control. Expression of other Bcl-2 family members is shown in Figure 2b. Error bars indicate SD.

Figure S5: Expression of Bcl-2 family proteins in *MLL-AF9* AMLs. Western blot analysis of expression of Bcl-2 family proteins in spleens of sick primary reconstituted mice. Panels indicate 8 separate gels, each of which included WT/*MLL-AF9* #1411 as a reference sample. The genotypes of fetal liver cells infected with *MLL-AF9* virus are indicated above the panels, as is the tumor identification number; broken lines separate AMLs of different genotypes. A1 controls were WEHI-231 cells (+) and WEHI-231 cells treated with cycloheximide (20 μg/mL for 1 h) (-). The upper four gels included a known Puma-positive control (293T cells constitutively expressing mouse puma protein) and the lower four gels included a known Noxa-positive control (tumor 1592). Noxa protein is the lower of the two bands shown, the upper band is non-specific. Hsp70 and Actin served as loading controls. Solid lines indicates electronic removal of a lane.

Figure S6: Expression of Bcl-2 family members following treatment. Expression of Bcl-2 family members in cultured primary *MLL-AF9* AMLs following treatment with cytotoxic agents. Western blot analysis of protein expression in WT/*MLL-AF9*

#1411 and *bmf*$^{-/-}$/MLL-AF9 #1415 after 6 h treatment with the indicated drugs. AML cell lines maintained in culture in IMDM with 10% FCS and supplemental IL-3 (see Materials and Methods) were treated with 600 ng/ml cytarabine, 25 nM bortezomib, 200 ng/ml daunorubicin, 300 ng/ml etoposide, either alone or in combination with 1 μg/ml ABT-737 as indicated, in presence of the pan-caspase inhibitor QVD (25 μM). NA is not available. Molecular weight markers (kD) are indicated.

Figure S7: Expression of Bcl-2 family members in WT and BH3-gene KO *MLL-AF9* AML cell lines following drug treatment. Bone marrow-derived AML cell lines cultured in IMDM with 10% FCS and IL-3 were treated with the indicated drugs and the pan-caspase inhibitor QVD (see materials and methods) for 3 h prior to harvesting for RNA analysis by qPCR. Δ Δ CT values normalized to HMBS control and made relative to cells treated with QVD only to determine drug-induced fold change. Data represents mean fold change ± SEM, with solid line indicating a value of 1. *MLL-AF9* AMLs analyzed by qPCR were: WT #1300, 1411, 1601; *bim*$^{-/-}$ #1158, 1249; *puma*$^{-/-}$ #1215, 1229; *noxa*$^{-/-}$ #1313, 1310; *puma*$^{-/-}$ *noxa*$^{-/-}$ #1443, 1444, 1445; *noxa*$^{-/-}$ *bmf*$^{/-}$ #1598, 1597; *puma*$^{-/-}$ *bim*$^{-/-}$ #1590, 1593.

Figure S8: Survival of mice injected with WT and BH3-gene KO *MLL-AF9* AMLs and treated with daunorubicin. Kaplan-Meier curves for mice bearing AML and treated with 5 mg/kg daunorubicin intravenously on days 1, 4 and 9 and either or ABT-737 (75 mg/kg) or vehicle intraperitoneally on days 1-5 and 8-12. Results are shown for individual tumors that were compiled in Figure 5. Transplanted mice were monitored daily for symptoms of AML and euthanized if morbidly ill or at the end of

experiment (100 days post treatment start). WT/*MLL-AF9* #1211, 1223, 1224, 1411 and 1414, *bim*$^{-/-}$ *MLL-AF9* #1158, 1213, 1249 and 1250 *puma*$^{-/-}$ *MLL-AF9* #1218, 1225, 1226 and 1229 and *noxa*$^{-/-}$ *MLL-AF9* #1306,1308, 1309, 1310 and 1311.

Figure S1

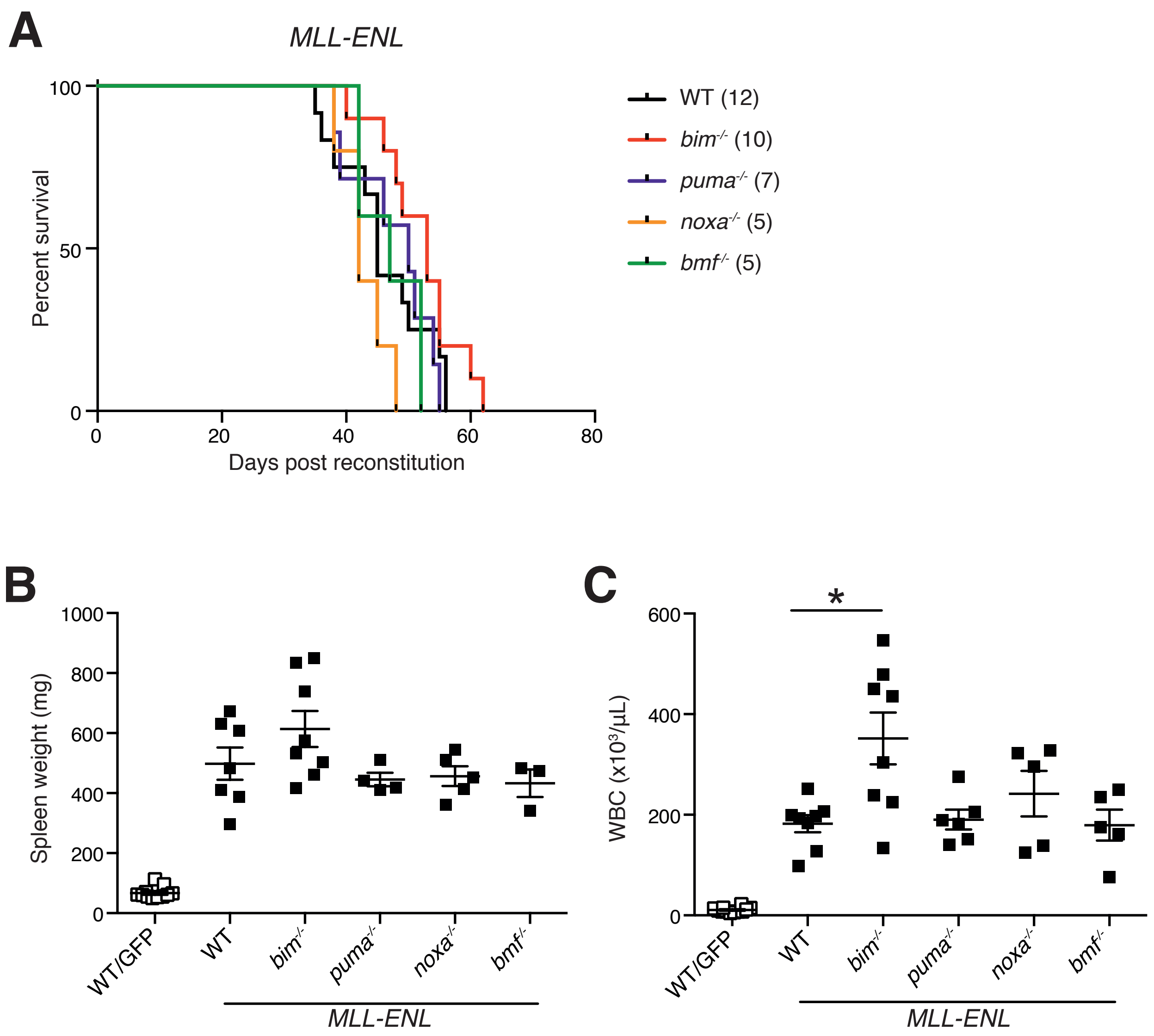
A
MLL-ENL
100
50
0
Percent survival
0
20
40
60
80
Days post reconstitution
WT (12)
bim-/- (10)
puma-/- (7)
noxa-/- (5)
bmf-/- (5)
B
1000
800
600
400
200
0
Spleen weight (mg)
WT/GFP
WT
bim-/-
puma-/-
noxa-/-
bmf-/-
MLL-ENL
C
*
600
400
200
0
WBC (x10³/µL)
WT/GFP
WT
bim-/-
puma-/-
noxa-/-
bmf-/-
MLL-ENL

Figure S2

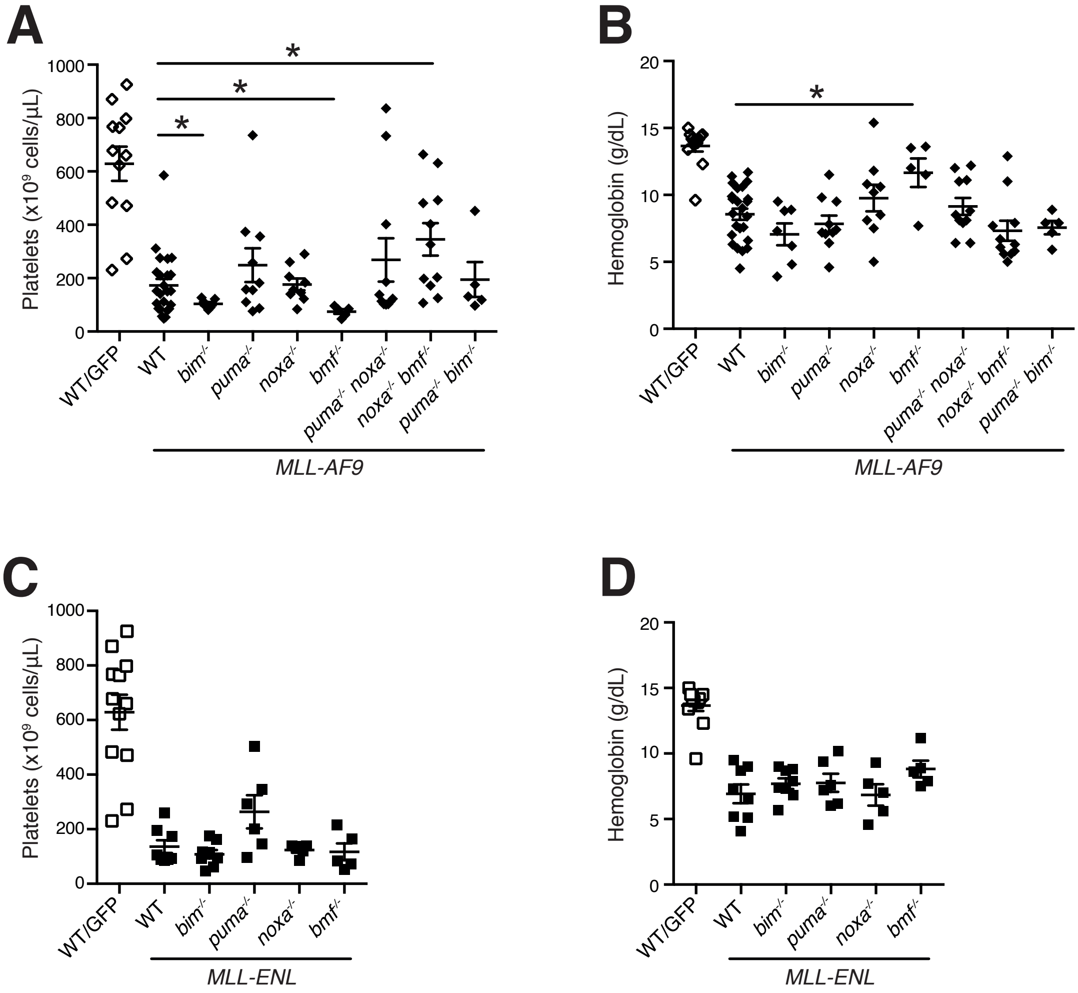

A
B
C
D
Platelets (x10⁹ cells/µL)
Hemoglobin (g/dL)
*
WT/GFP
WT
bim-/-
puma-/-
noxa-/-
bmf-/-
puma-/- noxa-/-
noxa-/- bmf-/-
puma-/- bim-/-
MLL-AF9
MLL-ENL

Figure S3

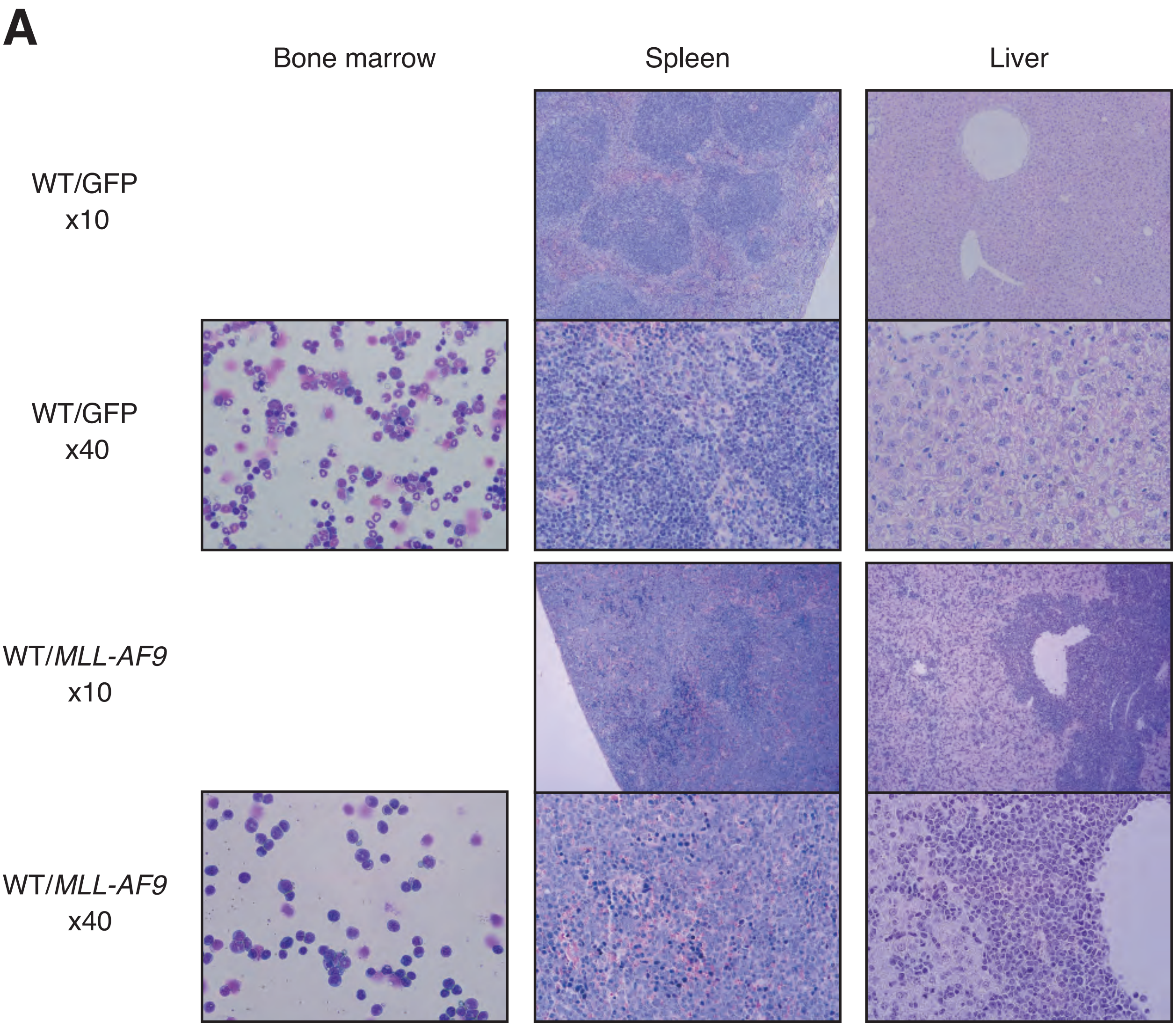
A
Bone marrow
Spleen
Liver
WT/GFP
x10
WT/GFP
x40
WT/*MLL-AF9*
x10
WT/*MLL-AF9*
x40

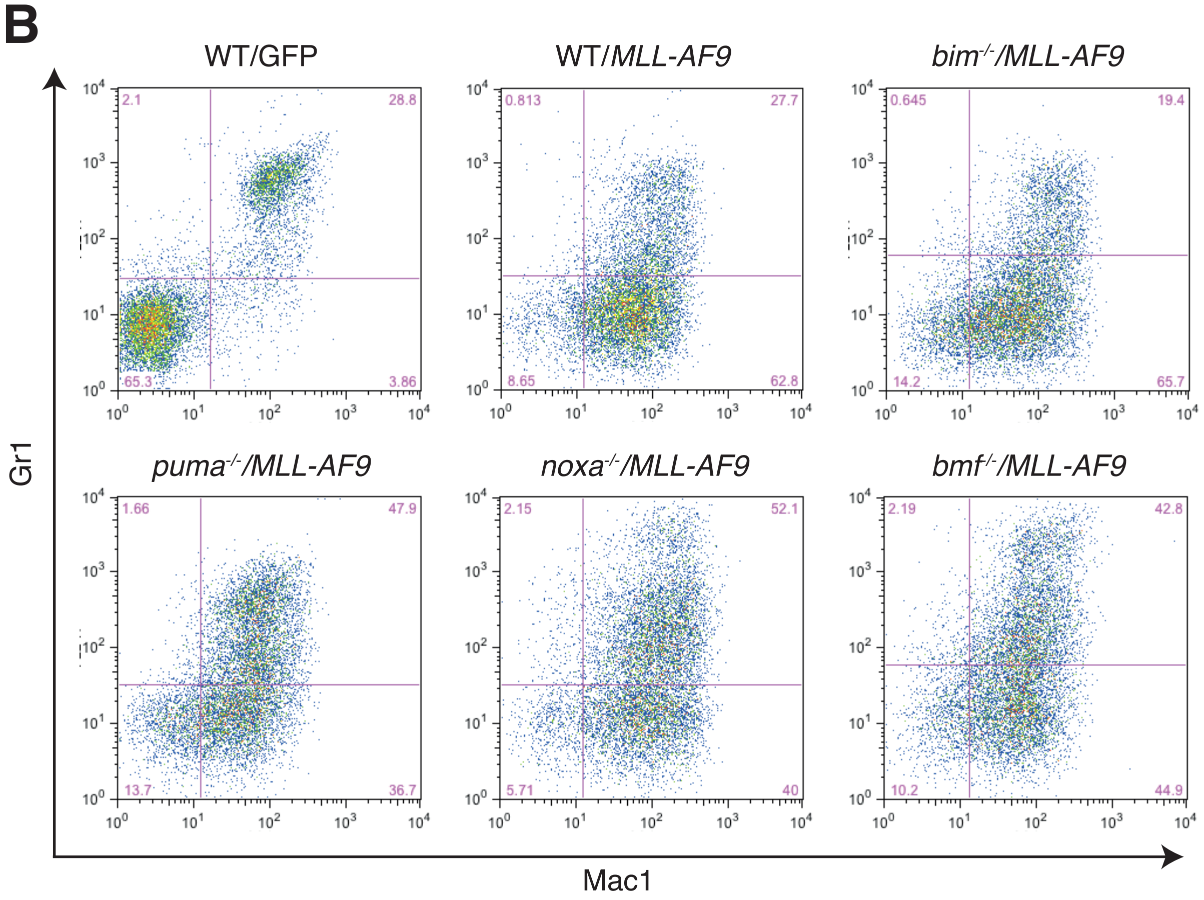
B
WT/GFP
2.1
28.8
65.3
3.86
WT/*MLL-AF9*
0.813
27.7
8.65
62.8
bim-/-/*MLL-AF9*
0.645
19.4
14.2
65.7
puma-/-/*MLL-AF9*
1.66
47.9
13.7
36.7
noxa-/-/*MLL-AF9*
2.15
52.1
5.71
40
bmf-/-/*MLL-AF9*
2.19
42.8
10.2
44.9
Gr1
Mac1

Figure S4

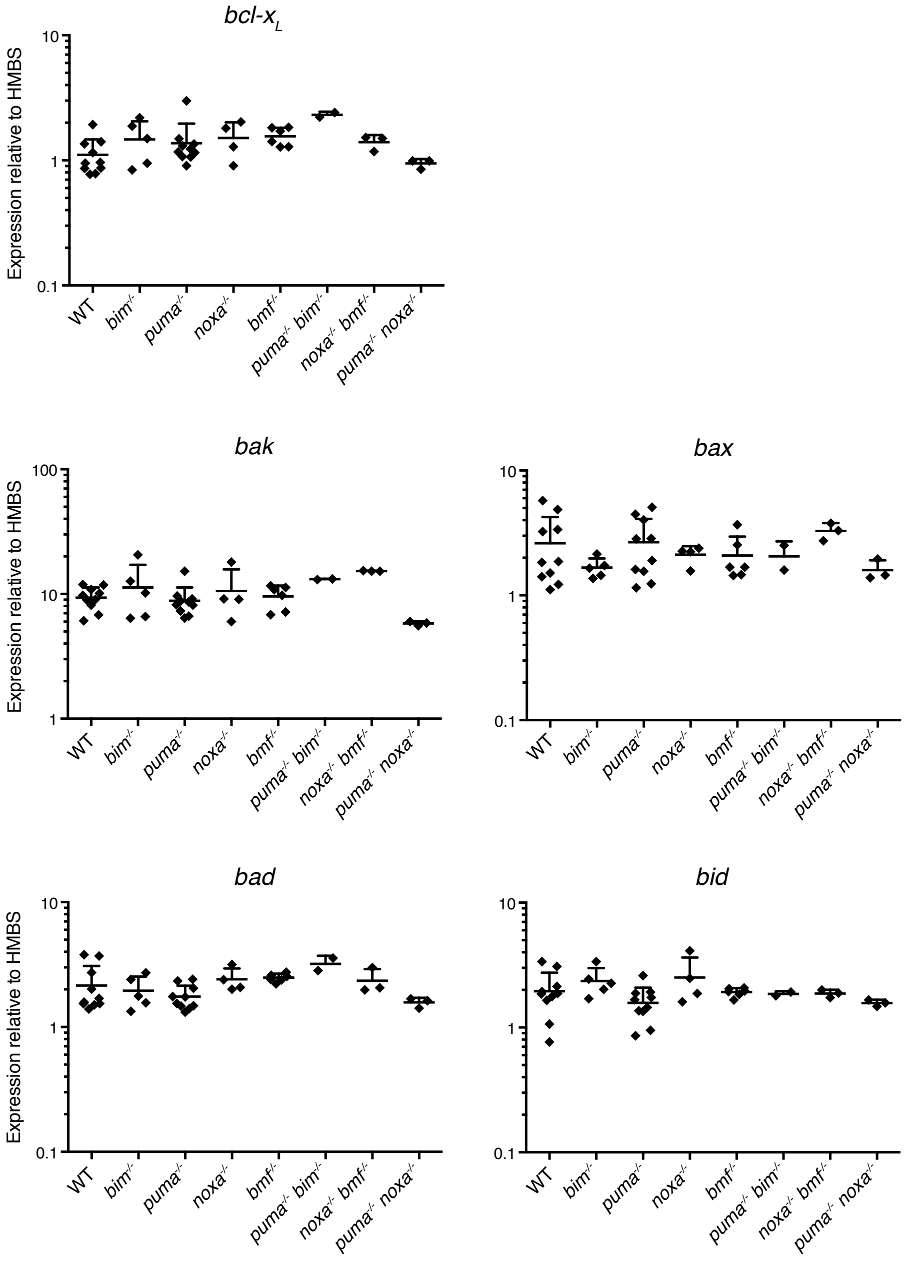

bcl-xL
bak
bax
bad
bid
Expression relative to HMBS
100
10
1
0.1
WT
bim-/-
puma-/-
noxa-/-
bmf-/-
puma-/- bim-/-
noxa-/- bmf-/-
puma-/- noxa-/-

Figure S5

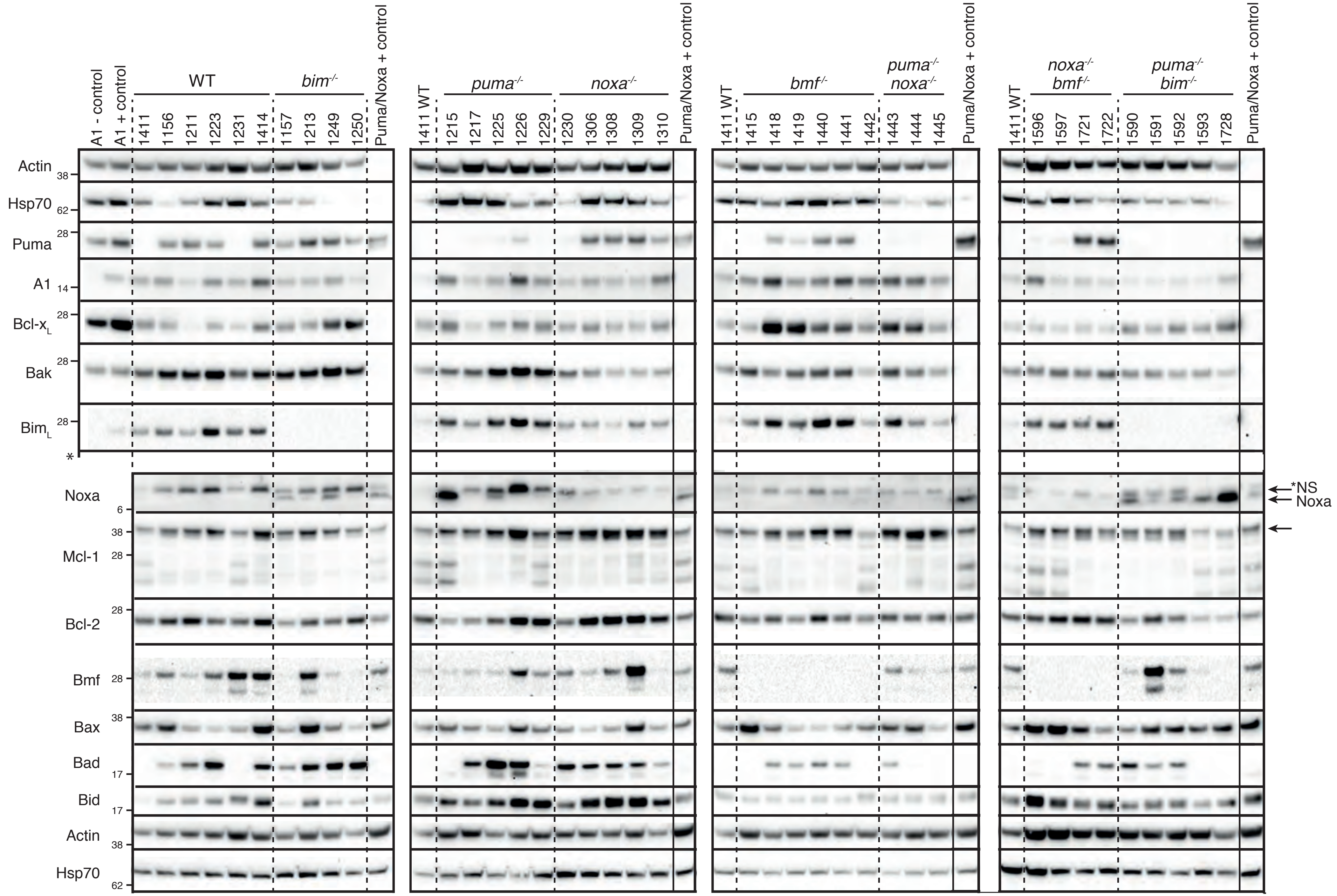

A1 - control
A1 + control
WT
1411
1156
1211
1223
1231
1414
bim-/-
1157
1213
1249
1250
Puma/Noxa + control
1411 WT
puma-/-
1215
1217
1225
1226
1229
noxa-/-
1230
1306
1308
1309
1310
bmf-/-
1415
1418
1419
1440
1441
1442
puma-/- noxa-/-
1443
1444
1445
noxa-/- bmf-/-
1596
1597
1721
1722
puma-/- bim-/-
1590
1591
1592
1593
1728
Actin
Hsp70
Puma
A1
Bcl-xL
Bak
BimL
*
Noxa
Mcl-1
Bcl-2
Bmf
Bax
Bad
Bid
38
62
28
14
6
17
*NS

Figure S6

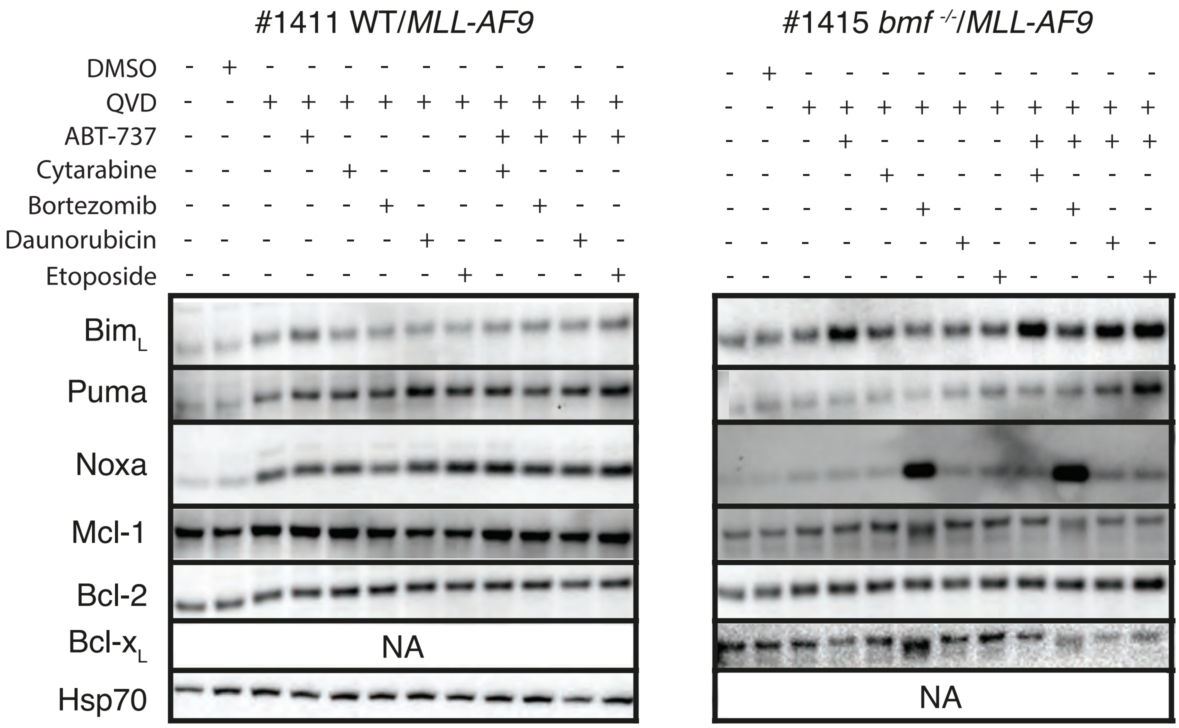

#1411 WT/MLL-AF9
#1415 bmf -/-/MLL-AF9
DMSO
QVD
ABT-737
Cytarabine
Bortezomib
Daunorubicin
Etoposide
BimL
Puma
Noxa
Mcl-1
Bcl-2
Bcl-xL
Hsp70
NA
NA

Figure S7

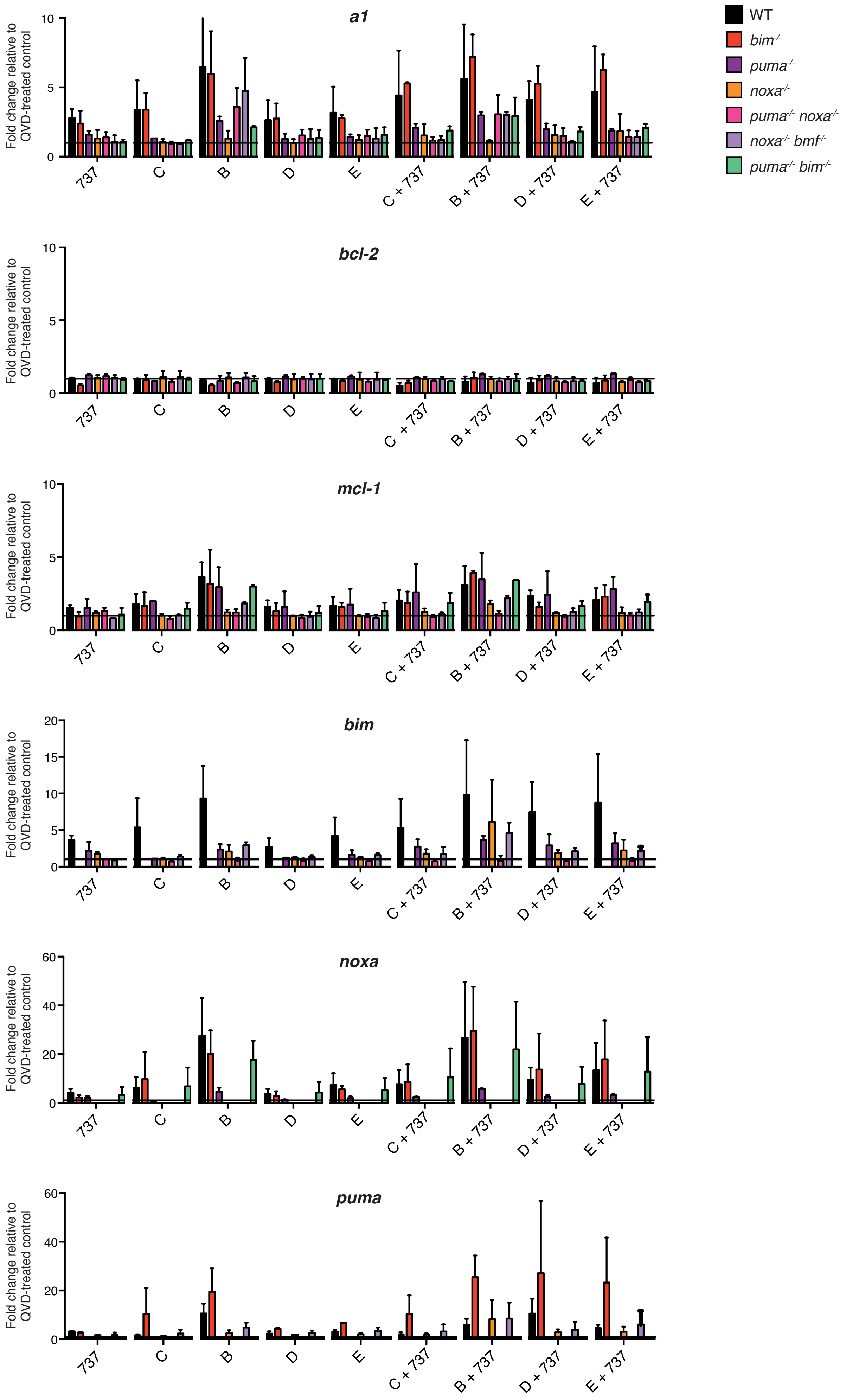

WT
bim-/-
puma-/-
noxa-/-
puma-/- noxa-/-
noxa-/- bmf-/-
puma-/- bim-/-
a1
bcl-2
mcl-1
bim
noxa
puma
Fold change relative to QVD-treated control
737
C
B
D
E
C + 737
B + 737
D + 737
E + 737

Figure S8

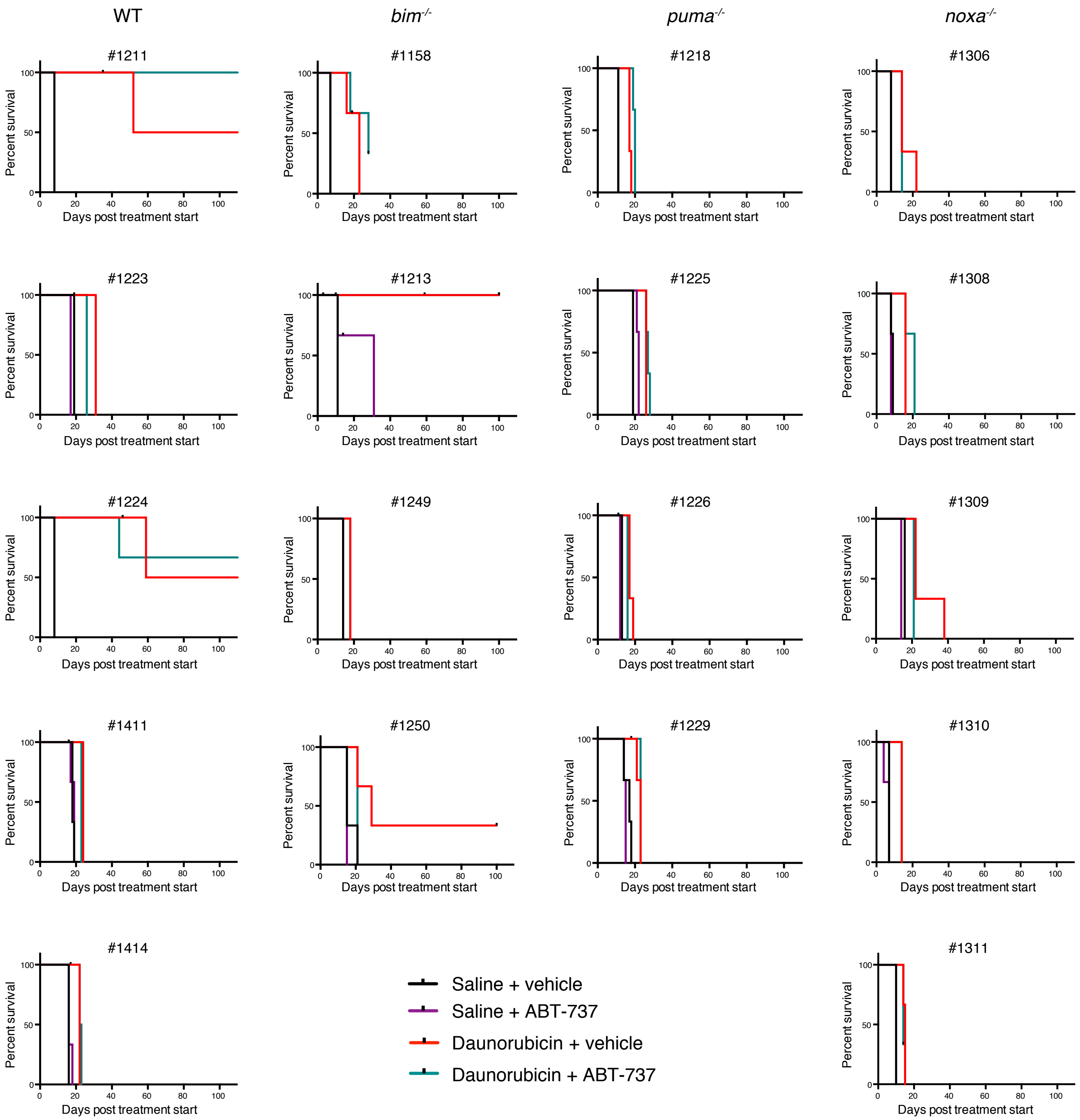
WT
bim-/-
puma-/-
noxa-/-
#1211
#1158
#1218
#1306
#1223
#1213
#1225
#1308
#1224
#1249
#1226
#1309
#1411
#1250
#1229
#1310
#1414
#1311
Percent survival
Days post treatment start
Saline + vehicle
Saline + ABT-737
Daunorubicin + vehicle
Daunorubicin + ABT-737
